# Polymerization-induced thermal self-assembly (PITSA)†
†Electronic supplementary information (ESI) available: Experimental details, characterization data, and additional figures. See DOI: 10.1039/c4sc03334e



**DOI:** 10.1039/c4sc03334e

**Published:** 2014-11-14

**Authors:** C. Adrian Figg, Alexandre Simula, Kalkidan A. Gebre, Bryan S. Tucker, David M. Haddleton, Brent S. Sumerlin

**Affiliations:** a George & Josephine Butler Polymer Research Laboratory , Center for Macromolecular Science & Engineering , Department of Chemistry , University of Florida , PO Box 117200 , Gainesville , FL 32611-7200 , USA . Email: sumerlin@chem.ufl.edu; b Department of Chemistry , University of Warwick , Coventry CV4 7AL , UK

## Abstract

Polymerization-induced self-assembly (PISA) is a versatile technique to achieve a wide range of polymeric nanoparticle morphologies. Most previous examples of self-assembled soft nanoparticle synthesis by PISA rely on a growing solvophobic polymer block that leads to changes in nanoparticle architecture during polymerization in a selective solvent. However, synthesis of block copolymers with a growing stimuli-responsive block to form various nanoparticle shapes has yet to be reported. This new concept using thermoresponsive polymers is termed polymerization-induced thermal self-assembly (PITSA). A reversible addition-fragmentation chain transfer (RAFT) polymerization of *N*-isopropylacrylamide from a hydrophilic chain transfer agent composed of *N*,*N*-dimethylacrylamide and acrylic acid was carried out in water above the known lower critical solution temperature (LCST) of poly(*N*-isopropylacrylamide) (PNIPAm). After reaching a certain chain length, the growing PNIPAm self-assembled, as induced by the LCST, into block copolymer aggregates within which dispersion polymerization continued. To characterize the nanoparticles at ambient temperatures without their dissolution, the particles were crosslinked immediately following polymerization at elevated temperatures *via* the reaction of the acid groups with a diamine in the presence of a carbodiimide. Size exclusion chromatography was used to evaluate the unimer molecular weight distributions and reaction kinetics. Dynamic light scattering and transmission electron microscopy provided insight into the size and morphologies of the nanoparticles. The resulting block copolymers formed polymeric nanoparticles with a range of morphologies (*e.g.*, micelles, worms, and vesicles), which were a function of the PNIPAm block length.

## Abstract 2

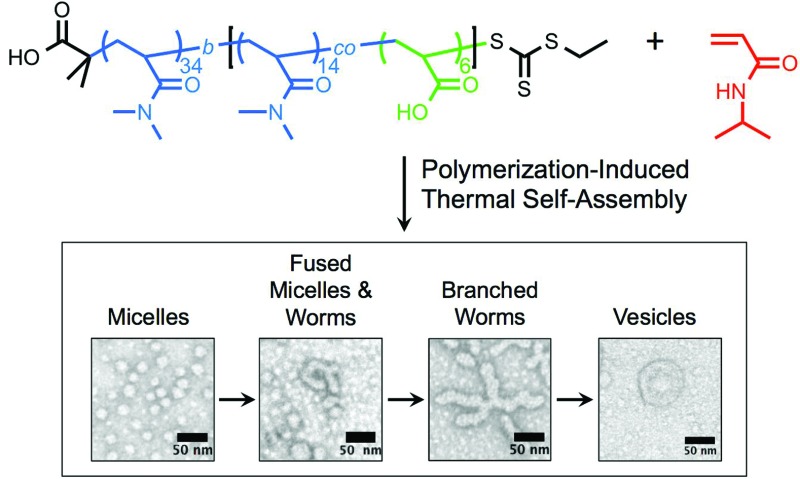
Polymerization-induced thermal self-assembly (PITSA) was conducted using thermoresponsive poly(*N*-isopropylacrylamide) to result in micelle, worm, and vesicle polymeric morphologies.

## Introduction

Self-assembled amphiphilic polymer nanoparticles have garnered substantial attention as potential delivery vehicles due to high stability at low solution concentrations, molecular encapsulation capabilities, and biocompatability.^1–3^ Micelles are perhaps the most exploited self-assembled polymer morphology because of their facile preparation and the ease with which they can be loaded.^4,5^ However, other types of self-assembled block copolymer morphologies, such as cylindrical micelles (*i.e.*, worms and nanorods) and vesicles, can have various advantages over the simple micellar structure. For example, in many cases polymeric worms and nanorods have higher loading capacities than micelles, though their solution properties, such as *in vivo* cell absorption, are not completely understood.^6^ Vesicles, or polymersomes, are able to encapsulate molecules in both their hydrophobic vesicle membrane and their hydrophilic interiors, providing the possibility of dual-component delivery.^7,8^

Although self-assembled polymer nanoparticles show increasing viability as delivery vehicles, inefficient and laborious preparation techniques may limit their broad application. Typically, the desired morphology that results from block copolymer assembly is developed through an often tedious bottom up approach at low concentrations (<5 w/w% solids).^9,10^ This strategy can entail multiple iterations of polymer synthesis, purification, and self-assembly *via* either solvent switching or film rehydration. The aggregated morphology is dictated primarily by the interfacial curvature within the aggregate and is a function of the packing parameter (*p*) described in eqn (1):1
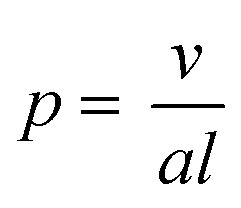
where *v* is the volume of the hydrophobic chain, *a* is the optimal area of the hydrophilic head group, and *l* is the length of the hydrophobic chain.^11^ A lower packing parameter (*p* ≤ 1/3) is indicative of high interfacial curvature and leads to spherical micelles, while worms and vesicles are observed as the packing parameter increases (1/3 < *p* < 1/2 and *p* ≤ 1/2, respectively).^12^ However, another factor that controls whether micelles, worms, vesicles, or higher order nanoparticles are formed is the method and finesse in which they are synthesized, processed, and assembled.^13^ This often leads to arduous experimental optimization, opportunities for errors through the multi-step preparation process, and difficulty scaling up nanoparticle yields.

Recent advances in the area of polymerization-induced self-assembly (PISA) have offered an alternative route to facilitate the preparation of assembled polymer nanoparticles.^14–21^ During PISA, various nanoparticle morphologies are obtained by the gradual *in situ* rearrangement of growing solvophobic chains and interparticle collisions in relatively concentrated solutions (up to 50 w/w% solids). These collisions allow the growing solvophobes to rearrange into lower energy morphologies as the hydrophobic segments grow to occupy larger volumes relative to the hydrophilic stabilizing block. This rearrangement leads to an increasing chain packing parameter and a morphological transition from micelles to worms to vesicles during polymerization.

Reversible-deactivation radical polymerization (RDRP) methods provide the control over polymer molecular weight needed to induce the gradual evolution of well-defined nanostructures during polymerization.^22–24^ Nitroxide-mediated polymerization (NMP) and atom transfer radical polymerization (ATRP) have been successfully employed for PISA;^25–28^ however, the majority of PISA reports rely on reversible addition-fragmentation chain transfer (RAFT) polymerization, due to the mild reaction conditions and functional group tolerance.^29^ Thus far, purely (and permanently) solvophobic polymers have been prepared by PISA *via* RAFT, including the synthesis of polystyrene in alcohols,^30–34^ poly(2-methoxyethyl acrylate) in water,^35^ poly(2-hydroxypropyl methacrylate) (PHPMA) in water,^36^ and nucleobase-containing polymers in chloroform and 1,4-dioxane.^37^

Imparting stimuli-sensitivity introduces an interesting characteristic to amphiphilic nanoparticles, bestowing “smart” behavior that can induce a phase transition from hydrophilic to hydrophobic under specific stimulus.^38^ Thermoresponsive polymers,^39^ for example, poly(*N*-isopropylacrylamide) (PNIPAm), are particularly attractive as their lower critical solution temperature (LCST) can be tailored by varying molecular weight,^40^ incorporating comonomers,^41,42^ or functionalizing end-group moieties.^5,43–46^ Recently, Zhong and co-workers showed that temperature responsive vesicles can be prepared by dissolving a PEG-*b*-poly(acrylic acid)-*b*-PNIPAm) copolymer in water above the LCST of PNIPAm with subsequent crosslinking of the acid groups.^47^ Monteiro and co-workers demonstrated a temperature directed morphological transformation by synthesizing a PNIPAm-*b*-polystyrene copolymer with a RAFT nanoreactor technique with induced phase separations *via* a variation of the solubility of the styrene core.^48^ Stucky and co-workers reported temperature responsive polymer nanoparticles from a precipitation polymerization of NIPAm and subsequent diacrylamide core crosslinking.^49^ An and co-workers prepared thermoresponsive PNIPAm nanogels *via* a diacrylate core crosslinking comonomer,^50^ while Zhang and co-workers reported multiple PNIPAm copolymers by dispersion polymerization.^51,52^ Armes and co-workers have exploited thermoresponsive behavior to tune the morphology of PHPMA-containing block copolymer nanoparticles synthesized by PISA.^53,54^

**Fig. 1:**
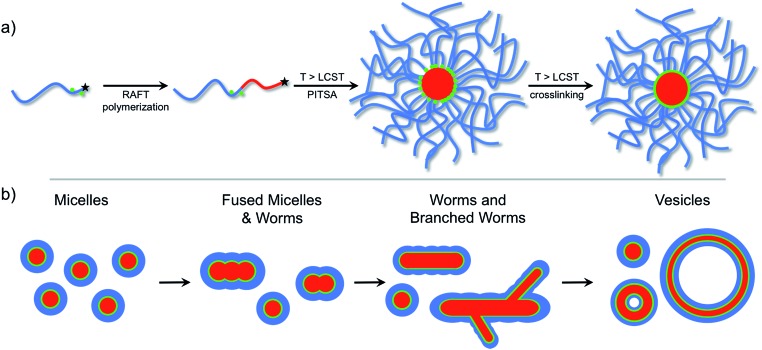
(a) Reversible addition-fragmentation chain transfer (RAFT) polymerization chain-extension of a hydrophilic macro chain transfer agent at 70 °C resulted in different self-assembled polymeric nanoparticle morphologies that were subsequently crosslinked. (b) Progression of polymer nanoparticle morphology with increasing hydrophobic polymer degree of polymerization.

The versatility of PISA, especially in aqueous media,^55^ offers substantial promise for facile soft nanoparticle synthesis.^56^ However, as far as we are aware, taking advantage of the *reversible* phase transition of stimuli-responsive polymers for self-assembly during PISA has not been reported. Herein, we report the thermally-triggered aggregation and reorganization of a growing NIPAm polymer to form diverse self-assembled polymer morphologies that depend on the PNIPAm degree of polymerization. We refer to this approach as polymerization-induced thermal self-assembly (PITSA) and believe that it offers considerable promise for the facile synthesis of responsive polymer nanoparticles. To do this, a hydrophilic RAFT macro chain transfer agent (macro-CTA) composed of primarily *N*,*N*-dimethylacrylamide (DMA) was chain extended with NIPAm in water at 70 °C (Fig. 1a). By performing the chain extension above the LCST of PNIPAm, the growing PNIPAm blocks self-assembled as they became increasingly insoluble. As the growth of the PNIPAm continued, the observed morphology of the self-assembled aggregates evolved from micelles, to fused micelles/worm-like micelles, to branched worms, and eventually to vesicles (Fig. 1b). As opposed to previous reports of PISA that led to aggregates that were readily characterized due to the stability provided by the solvophobic block, the assemblies prepared by PITSA are susceptible to dissociation on cooling. Therefore, to aid with characterization of the nanoparticles, we included a small fraction of acrylic acid (AA) units within the hydrophilic PDMA block to allow subsequent crosslinking with ethylenediamine.^47^ The resulting polymer self-assemblies were thus stable and more readily characterized.

## Results and discussion

**Scheme 1:**
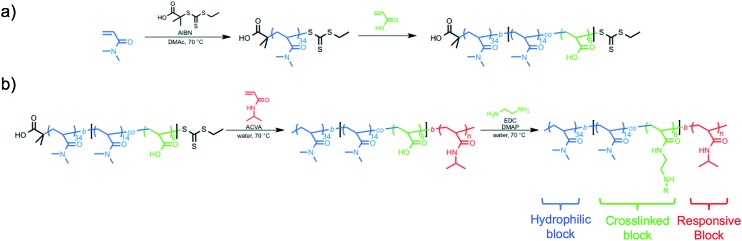
Synthesis of thermoresponsive block copolymers (a) macro chain transfer agent synthesis of polyDMA_34_-*b*-poly(DMA_14_-*co*-AA_6_) by RAFT (co)polymerization of *N*,*N*-dimethylacrylamide (DMA) and acrylic acid (AA) (b) RAFT chain-extension using *N*-isopropylacrylamide and subsequent crosslinking.

The hydrophilic macro-CTA was synthesized by the homogeneous polymerization of DMA in DMAc at 70 °C with sequential addition of AA. DMA conversion was monitored by ^1^H NMR spectroscopy, and when 32% of monomer was consumed, AA was added to the reaction vessel (Scheme 1a). The polymerization was quenched at an overall DMA conversion of 52%, and end-group analysis of the purified polymer revealed a final composition of polyDMA_34_-*b*-poly(DMA_14_-*co*-AA_6_) (Fig. S1†). Size exclusion chromatography (SEC) equipped with a multi-angle light scattering (MALS) detector showed a unimodal and symmetric molecular weight distribution with an absolute *M*_n_ of 5690 g mol^–1^ (*Đ*_M_ = 1.05) (Fig. S2†).

Chain-extension polymerizations with varying feed ratios of NIPAm were carried out to investigate how the degree of polymerization of PNIPAm affected the self-assembly of the nanoparticles. For brevity, only four of the block copolymers with varying degrees of polymerization of the PNIPAm block will be discussed in detail, though the characterization results for all other block copolymers prepared are included in ESI.† Each polymerization was conducted at 15 w/w% solids in water at 70 °C for 3 h, at which point monomer conversions had reached between 82–99% by ^1^H NMR spectroscopy. The variance in monomer conversions could be attributed to the varying [monomer] : [CTA] : [initiator] ratios. By conducting the chain extension above the LCST of the growing PNIPAm block, the developing block copolymers self-assembled during polymerization, which was apparent from the reaction mixture gradually transitioning from homogeneous to heterogeneous as conversion increased. The viscosity of the solutions appeared to reach a maximum at the intermediate PNIPAm *DP*_n,theo_ presumably from worm entanglements.^50^ The polymerizations were quenched while holding the temperature above 70 °C, ensuring the newly formed nanoparticle morphologies remained intact.

**Fig. 2:**
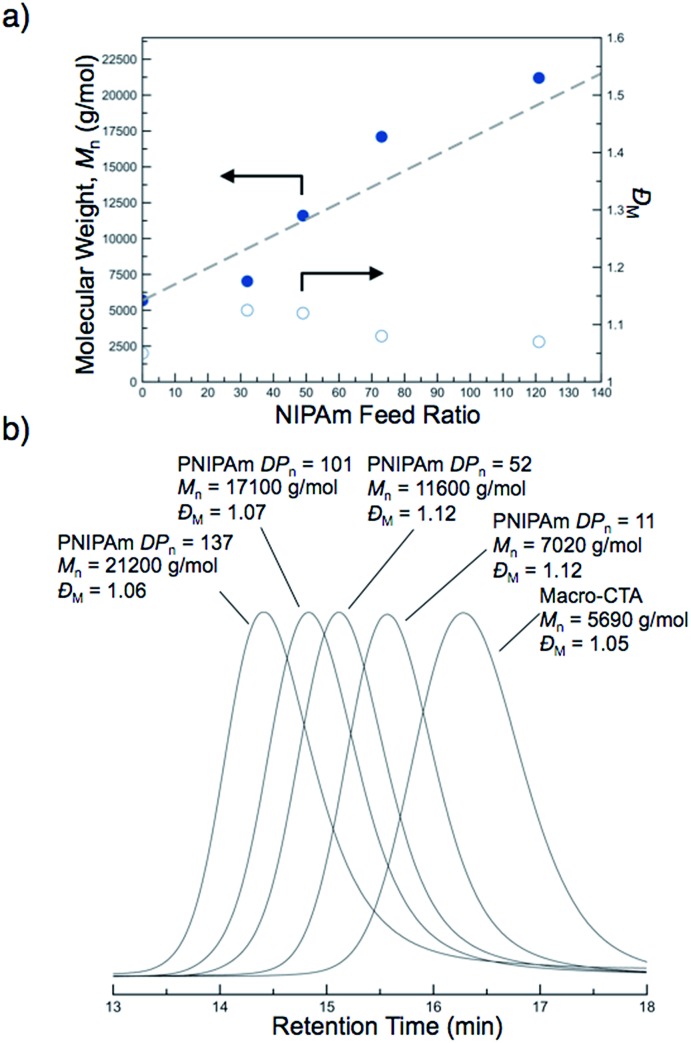
Molecular weight evolution during the polymerization-induced thermal self-assembly (PITSA) of polyDMA_34_-*b*-poly(DMA_14_-*co*-AA_6_)-*b*-polyNIPAm_*n*_ by the chain extension of a macro chain transfer agent containing *N*,*N*-dimethylacrylamide (DMA) and acrylic acid (AA), polyDMA_34_-*b*-poly(DMA_14_-*co*-AA_6_), with *N*-isopropylacrylamide (NIPAm). (a) Absolute number-average molecular weight (*M*_n_) as a function of NIPAm feed ratio (b) SEC chromatograms as a function of PNIPAm degree of polymerization (PNIPAm *DP*_n_).

The molecular weights of the block copolymers were determined by taking an aliquot from the polymerization mixture, purifying *via* dialysis, and conducting SEC-MALS. A linear increase in the absolute molecular weight was observed with increasing NIPAm feed ratio (Fig. 2a). While traditional PISA often leads to polymers with somewhat broad molecular weight distributions and/or multimodal SEC traces with high molecular weight shoulders, all of the block copolymers formed by this new approach were unimodal and of low molar mass dispersity (*Đ*_M_ < 1.12) (Fig. 2b). These results suggest the block copolymers prepared by PITSA were well-defined and indicative of excellent blocking efficiency, even at high monomer conversions (Fig. 2b). The enhanced control observed here is potentially the result of a low concentration of radicals during polymerization and/or high mobility of the propagating chains within the nanoparticle cores due to the presence of water in the core, which can serve to plasticize the growing PNIPAm block.^57^

**Fig. 3:**
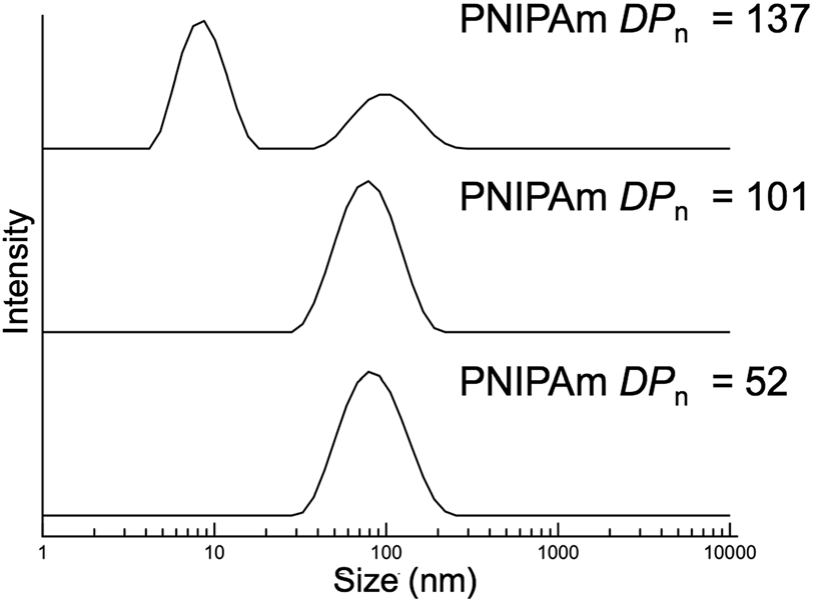
Dynamic light scattering measurements of purified nanoparticles with varying poly(*N*-isopropylacrylamide) degrees of polymerization (PNIPAm *DP*_n_).

The typical methods used to analyze block copolymer assemblies require either sample preparation or characterization at ambient temperature. Therefore, it was necessary to crosslink the nanoparticles formed during PITSA immediately after polymerization and prior to cooling. The pendant acid groups of the acrylic acid units in the hydrophilic shell of the nanoparticles were crosslinked by addition of ethylenediamine in the presence of 1-ethyl-3-(3-dimethylaminopropyl)carbodiimide (EDC) and catalytic 4-dimethylaminopyridine (DMAP) (Scheme 1b). These reactions were conducted at polymerization temperatures and immediately after the polymerizations were quenched by opening the reaction mixture to air. The polymerization solutions remained viscous after crosslinking, but subsequently changed from yellow to colorless due to aminolysis of the trithiocarbonate RAFT end group. Upon cooling to ambient temperature, the viscosity reduced, implying dissolution of some of the nanoparticles, likely due to the inherently low efficiency of EDC coupling in neutral water.^58^ Dynamic light scattering (DLS) analysis of the reaction mixtures showed distributions that could be attributed to the presence of both unimers and nanoparticles with larger hydrodynamic diameters (*D*_h_). To remove the unimer impurities, the nanoparticles were purified *via* dialysis and lyophilization (Fig. S17†). DLS measurements of the purified nanoparticles showed predominantly distributions of higher *D*_h_, suggesting primarily the larger nanoparticles remained (Fig. 3).

The nanoparticles with PNIPAm blocks of *DP*_n_ = 52, 101, and 137 resulted in large size distributions that might be expected of higher order assemblies, such as worms and vesicles. The resulting DLS histogram for the nanoparticles from the block copolymer with PNIPAm with *DP*_n_ of 137 showed a distribution that also contained a population of small particles, which might result from residual unimers or coupled chains. It should be noted that while these results indicate the presence of particles of a size range that might be expected for self-assembled block copolymers, the non-spherical and multimodal nature of these distributions suggests these sizes are approximate.

**Fig. 4:**
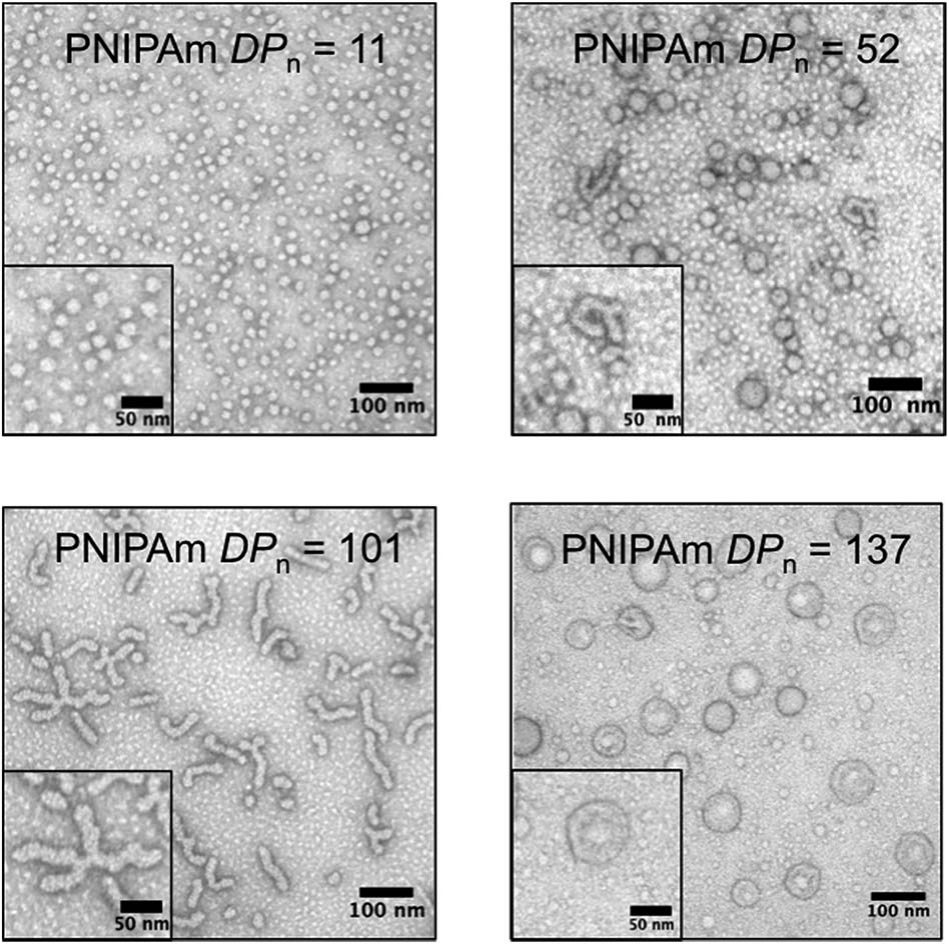
Transmission electron microscopy of the unpurified crosslinked polymer aggregates showing the progression of nanoparticle morphology as the poly(*N*-isopropylacrylamide) degree of polymerization (PNIPAm *DP*_n_) increased (2% uranyl acetate aqueous solution negative stain).

To gain further insight into the morphology of the block copolymer aggregates formed during the polymerization, transmission electron microscopy (TEM) was conducted after crosslinking of the nanoparticles formed *in situ* by reacting with ethylenediamine. As the PNIPAm *DP*_n_ increased, the predominant nanoparticle morphology changed. TEM images of the cooled reaction solution containing the crosslinked particles revealed multiple nanoparticle morphologies (Fig. 4). However, smaller particles (<10 nm) in the background were also present. These small features were attributed to residual polymeric impurities that arise from incomplete crosslinking and dead polymeric chains.^32^ Nevertheless, the majority of these impurities were successfully removed by dialysis, as evidenced by DLS and TEM (ESI†).

With a PNIPAm *DP*_n_ = 11, uniform micelles of approximately 20 nm diameter were obtained. The PNIPAm *DP*_n_ = 52 resulted in a mixture of micelles and fused micelles/worms. The observed micelles ranged in size from 20 to 40 nm, whereas multiple sizes of fused micelles and worms were observed. As the PNIPAm chains reached a *DP*_n_ = 101, predominantly worms and branched worms were observed, with minimal residual micelles being present. By increasing the *DP*_n_ of the PNIPAm to 137, structures that appeared to be vesicle-like were visible. The use of an osmium tetroxide positive stain suggested the presence of a bilayer showing a hollow core absent of polymeric material (ESI†). Combinations of the discussed polymeric morphologies were also observed in other examples of PITSA (ESI†).

**Fig. 5:**
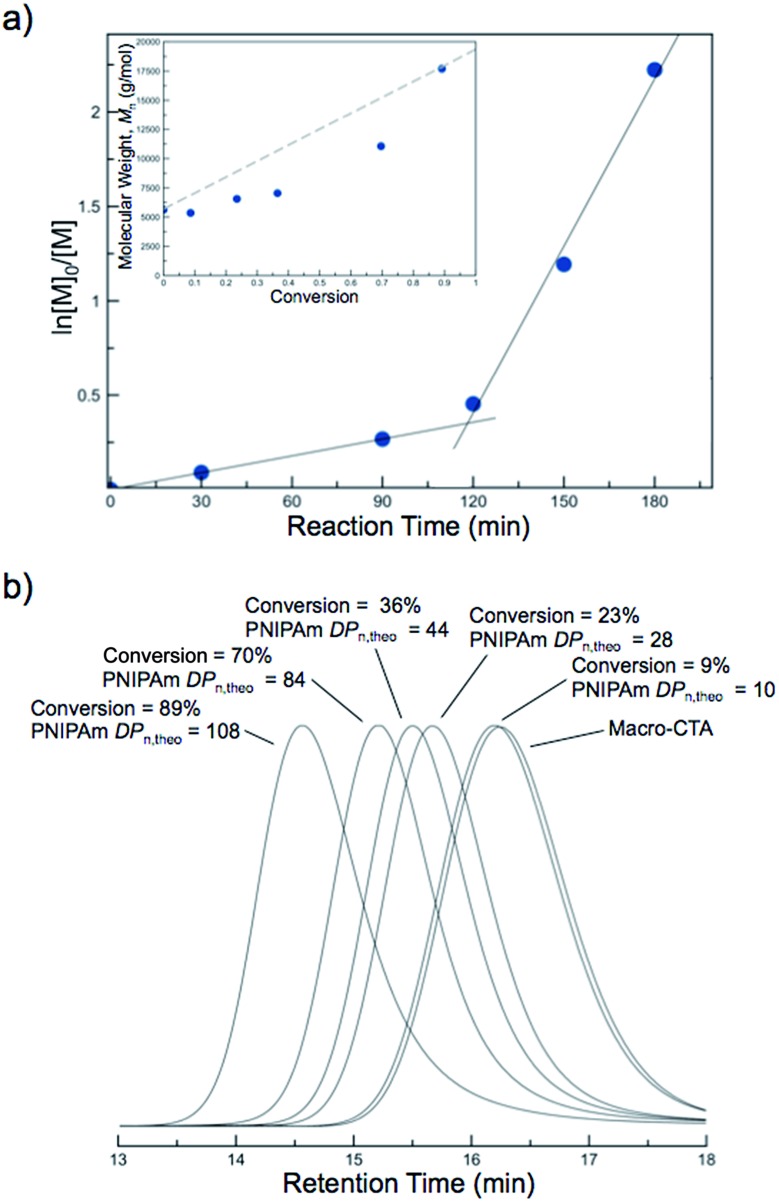
Data from the PITSA chain extension of a *N*,*N*-dimethylacrylamide (DMA) and acrylic acid (AA) containing macro chain transfer agent, [polyDMA_34_-*b*-poly(DMA_14_-*co*-AA_6_)], with *N*-isopropylacrylamide (NIPAm) at 70 °C. (a) First-order kinetics plot showing an increase in rate during the polymerization. Inset: *M*_n_ *vs.* conversion. (b) SEC traces showing unimodal distributions shifting to higher molecular weights as the polymerization proceeds.

Kinetic studies of the aqueous NIPAm chain extensions were conducted to gain further insight into the nature of the polymerization. Five separate polymerizations with identical stoichiometry ([NIPAm] : [polyDMA_34_-*b*-poly(DMA_14_-*co*-AA_6_) macro-CTA] : [I] = 121 : 1 : 0.04) were conducted at 70 °C. A separate polymerization was quenched every 30 min, and NIPAm conversion was monitored by ^1^H NMR spectroscopy. The first-order kinetic plots suggested a constant rate of polymerization for the first ∼120 min, after which the rate of polymerization appeared to increase (Fig. 5a). The observed increase in polymerization rate was also observed in previous PISA systems and is attributed to the self-assembly of growing solvophobic chains, which leads to an environment conducive to monomer solubilization.^56^ Once this occurs, the monomer is thought to swell the assembled regions of solvophobic chains. The high local monomer concentration that results from this leads to an increased rate of polymerization. Therefore, the point at which the change in rate was observed provides insight into the critical solution *DP*_n_ of the PNIPAm block at which self-assembly occurs at 70 °C. The evolution of *M*_n_ *versus* NIPAm conversion showed an approximately linear increase in molecular weight at low conversions, with slightly higher than expected molecular weights being present at elevated conversion (Fig. 5a, inset). The corresponding SEC traces shifted to higher molecular weights during the polymerization, with unimodal distributions and good macro-CTA blocking efficiency being apparent (Fig. 5b). Therefore, despite the induced nucleation of the PNIPAm into hydrophobic regimes, the polymerization still proceeded with good control over the molecular weight and dispersity while maintaining the mediating trithiocarbonate RAFT groups up to high monomer conversions.

## Conclusions

This report demonstrated for the first time polymerization-induced thermal self-assembly (PITSA), using the triggered hydrophobic self-assembly of PNIPAm moieties to induce nanoparticle reorganization during polymerization in an aqueous medium. PISA is typically limited to polymer/solvent combinations that allow monomer solubility and polymer insolubility in a permanently selective solvent. The PITSA approach reported here expands this highly useful process to allow for the preparation of responsive polymer nanoparticles by exploiting the temperature-sensitivity of PNIPAm in water. Given the variety of stimuli-responsive polymers that have been reported, this approach dramatically expands the library of possible PISA systems and introduces possible new approaches to synthesizing “smart” polymeric nanoparticle delivery vehicles using PITSA (“PITSA delivery”). Although our studies have focused exclusively on a thermoresponsive polymer with LCST-type behavior, this same general strategy should be applicable to temperature-sensitive polymers that demonstrate UCST behavior as well. Therefore, many other temperature-responsive polymers may prove to be viable slices of the PITSA pie.
