# Supplementary material for: Polymerization-induced thermal self-assembly (PITSA)
Source: Chem Sci. 2014 Nov 14;6(2):1230–6. doi: 10.1039/c4sc03334e (PMC5811124; doi:10.1039/c4sc03334e)
Supplement: Supplementary file 1 [file SC-006-C4SC03334E-s001.pdf]

Electronic Supplementary Material (ESI) for Chemical Science.
This journal is © The Royal Society of Chemistry 2014

Electronic Supplementary Information for

Polymerization-Induced Thermal Self-Assembly (PITSA)

C. Adrian Figg,[a] Alexandre Simula,[b] Kalkidan Asmelash Gebre,[a] Bryan S. Tucker,[a] David M. Haddleton, [b] and Brent S. Sumerlin*,[a]

[a]George & Josephine Butler Polymer Research Laboratory, Center for Macromolecular Science & Engineering, Department of Chemistry, University of Florida, PO Box 117200, Gainesville, FL 32611-7200

[b]Department of Chemistry, University of Warwick, Coventry CV4 7AL, United Kingdom

Experimental

Materials

4-Dimethylaminopyridine (DMAP, > 99%, Sigma-Aldrich), 4,4′-azobiscyanovaleric acid (ACVA, 98%, Alfa-Aesar), ethylenediamine (99%, Alfa Aesar), *N,N*-dimethylacetamide (DMAc, > 99%, Fisher Scientific), trioxane (> 99.5%, Acros Organics), 1-ethyl-3-(3-dimethylaminopropyl)carbodiimide•HCl (96%, Combi-Blocks) were used as received. *S*-Ethyl-*S*′-(α,α′-dimethyl-α″-acetic acid)-trithiocarbonate chain-transfer agent (CTA) was synthesized according to a previously published report.[1] 2,2′-Azobisisobutyrlnitrile (AIBN, 98%, Sigma-Aldrich) was recrystallized 3× from ethanol prior to use. *N*-Isopropylacrylamide (NIPAm, > 98%, TCI Chemicals) was recrystallized 3× from hexanes prior to use. *N,N*-Dimethylacrylamide (DMA, 99%, Sigma-Aldrich) and acrylic acid (AA, 99.5%, Alfa Aesar) were filtered through basic alumina prior to use.

Characterization

^{1}H NMR spectroscopy was conducted on an Inova 500 MHz, 2 RF channel instrument at 25 °C. Chloroform-*d* (Cambridge Isotopes Laboratories, Inc, 99.8%), DMSO-d_6 (Cambridge Isotopes Laboratories, Inc, 99.9%), and D_2O (Cambridge Isotopes Laboratories, Inc, 99.9%) solvents were used as received.

Size exclusion chromatography (SEC) was performed in dimethylacetamide (DMAc) with 50 mM LiCl at 50 °C and a flow rate of 1.0 mL min^{-1} (Agilent isocratic pump, degasser, and autosampler, colums: Plgel 5 μm guard

+ two ViscoGel I-series G3078 mixed bed columns: molecular weight range 0-20 × 10^3 and 1-100 × 10^4 g mol^{-1}). Detection consisted of a Wyatt Optilab T-rEX refractive index detector operating at 658 nm and a Wyatt miniDAWN Treos light scattering detector operating at 659 nm. Absolute molecular weights and polydispersities were calculated using the Wyatt ASTRA software and 100% mass recovery methods. Prior to absolute molecular weight determination, samples with NIPAm $DP_{n,theo}$ = 26, 49, 73, and 121 were purified via dialysis against DI water for 4 days (Spectra/Por 3 Dialysis Membranes (3500 MWCO) from Spectrum Laboratories) before being isolated by then freeze-drying.

Dynamic light scattering analysis was performed with a Malvern Zetasizer Nano ZS (Model No. ZEN 3600, Malvern Instruments Ltd., Worcestershire UK) at 25 °C. Purified samples were prepared by dialyzing against DI water for 7 days using Spectra/Por Float-A-Lyzer G2 100k MWCO 5 mL tubes from Spectrum Labs and subsequently freeze-dried. All samples were diluted to 5 mg/mL solutions and placed in a quartz low-volume cuvette for analysis.

Transmission electron microscopy was conducted on an H-700 from Hitachi High Technologies America, Inc., Schaumber, IL USA. Digital images were acquired with a Veleta 2k × 2k camera and iTEM software (Olympus Soft-Imaging Solutions Corp., Lakewood, CO). Electron Microscopy Sciences Formvar Carbon Film on 400 mesh nickel grids (FCF400-Ni) were used for all measurements. For unpurified samples, 10 μL polymerization solution sample diluted to 2.5 mg/mL was placed on a grid for 1 min. The excess solvent was wicked off and the grid air-dried. For staining, a drop of 2% uranyl acetate in water was placed on the grid and allowed to sit for 45 s. The excess solvent was wicked off and the grid air-dried. Purified samples from dialysis were diluted to 0.25 mg/mL, and TEM grids were prepared analogous to unpurified samples. For osmium tetroxide staining, a purified 0.125 mg/mL nanoparticle solution was placed on a grid for 1 min. The excess solvent was wicked off and the grid air-dried. The grid was then stained in an osmium tetroxide vapor chamber for 2 h.

Procedures

Synthesis of PDMA-AA macro chain-transfer agent

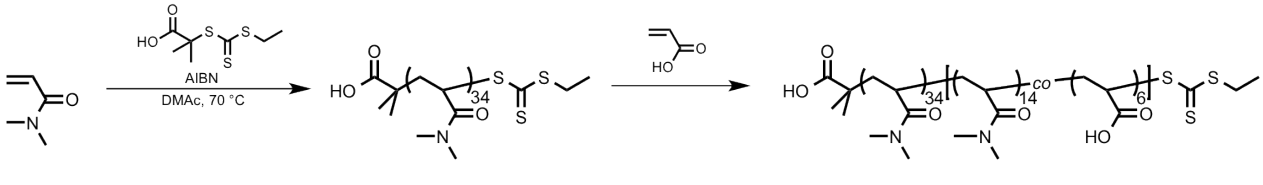

DMA (8.00 g, 80.8 mmol), CTA (181 mg, 0.808 mmol), and AIBN (6.60 mg, 0.404 mmol) were placed in a Schlenk flask to yield a [DMA]:[CTA]:[I] ratio = [100]:[1]:[0.05]. DMAc (20 mL) was added to make the monomer concentration 5 M, and 1,3,5-trioxane (120 mg) was added as an internal standard. The flask was sealed with a glass stopper, and a rubber septum was placed over the arm joint. The reaction was subject to 3 freeze-pump-thaw cycles, and left to stir at 70 °C. DMA conversion was monitored by ^{1}H NMR spectroscopy (chloroform-*d*, 500 MHz). Meanwhile, acrylic acid (1.75 g, 24.2 mmol) was purged with N_2 in a separate flask for 30 min. When the DMA polymerization had been stirring for 60 min (32% monomer conversion by ^{1}H NMR spectroscopy), the acrylic acid was cannulated into the reaction vessel. After 90 min (53% monomer conversion by ^{1}H NMR spectroscopy), the flask was removed from heat, opened to the atmosphere, and placed in a cold-water bath to stir. The solution was added drop-wise to cold diethylether to precipitate a yellow polymer. The polymer was subsequently re-precipitated into cold diethylether from THF and vacuum dried, yielding 4.07 g yellow powder. ^{1}H NMR spectroscopy end group analysis (DMSO-d_6, 500 MHz) allowed a comparison of the methylene peak next to the trithiocarbonate group (t, 1H, 3.61 ppm) to the acrylic acid proton (s, 11.5 – 12.5 ppm) and indicated a polymer composition of PDMA$_{48}$AA$_6$ ($M_{n,\mathrm{NMR}}$ = 5410 g/mol) while GPC$_{\mathrm{MALS}}$ absolute molecular weight analysis found an M_n = 5690 g/mol and $Đ_{\mathrm{M}}$ = 1.05. Final macro-CTA composition from conversion and end-group analysis: polyDMA$_{34}$-*b*-poly(DMA$_{14}$-*co*-AA$_6$).

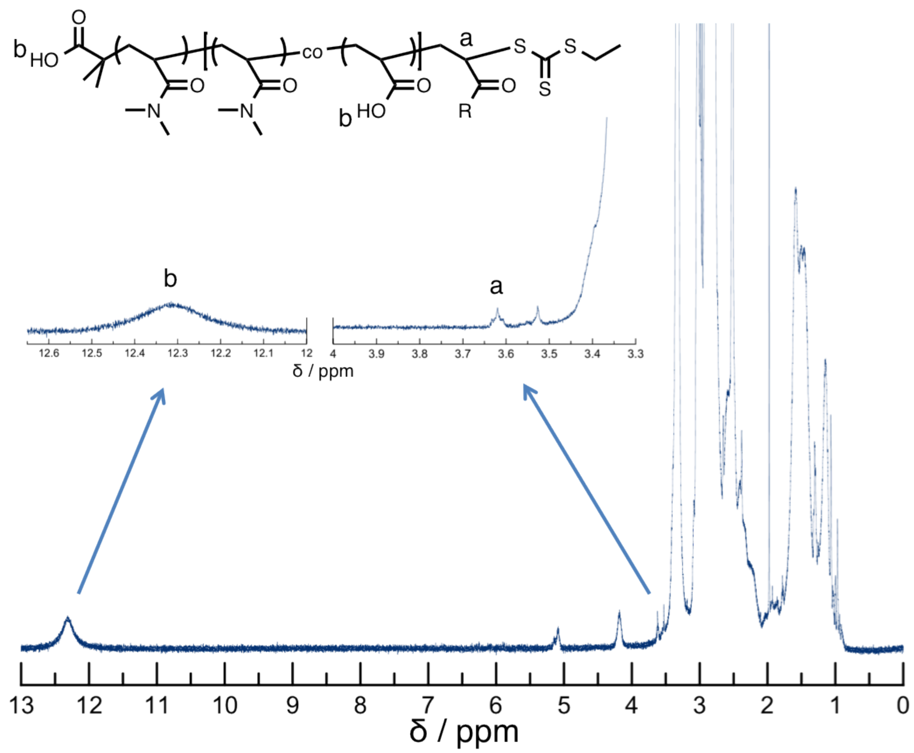

Fig. S1. ^{1}H NMR spectroscopy (DMSO-d_6, 500 MHz) for end-group analysis of polyDMA$_{34}$-*b*-poly(DMA$_{14}$-*co*-AA$_6$).

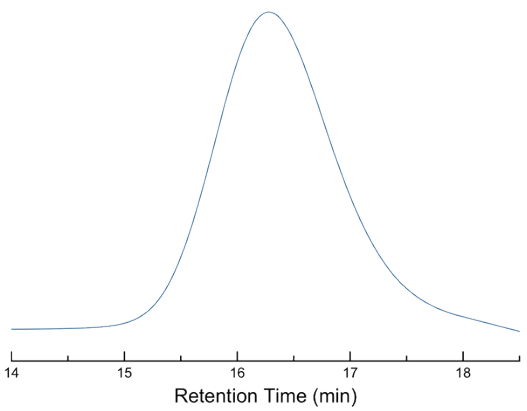

Fig. S2. SEC trace (DMAc eluent) of polyDMA$_{34}$-*b*-poly(DMA$_{14}$-*co*-AA$_6$) (M_n 5690 g/mol, $Đ_M$=1.05).

Nanoparticle synthesis with varying NIPAm feed ratio

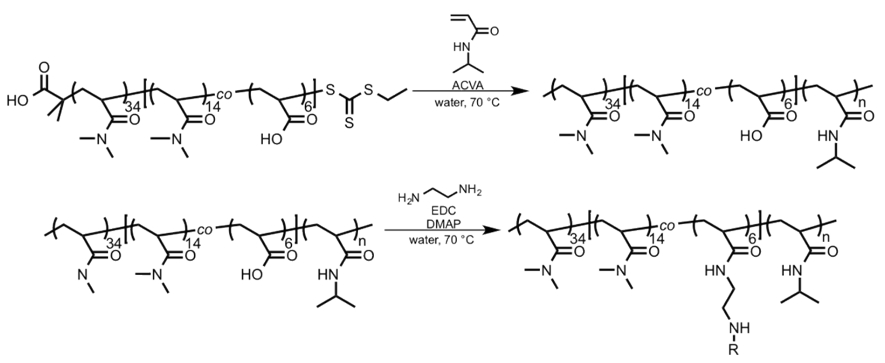

polyDMA$_{34}$-*b*-poly(DMA$_{14}$-*co*-AA$_6$)-*b*-polyNIPAm$_{26}$:

The macro-CTA polyDMA$_{34}$-*b*-poly(DMA$_{14}$-*co*-AA$_6$) (70.0 mg, 0.0123 mmol), NIPAm (45.2 mg, 0.400 mmol), ACVA (0.140 mg, 5.00 × 10^{-4} mmol, 1.00 mg/mL solution in DI water) were placed in a vial with a rubber septum to give a [NIPAm]:[CTA]:[I] ratio of [32]:[1]:[0.04]. DI water (0.654 g) was added to achieve a 15 w/w% solids concentration. The solution was deoxygenated by purging with N_2 for 30 min, then placed in a heating block at 70 °C. After 3 h, the vial was opened to the atmosphere, while still in the heating block, and subsequently stirred for 30 min to ensure reaction quenching. An aliquot (200. µL) of solution was taken for ^{1}H NMR spectroscopy (D_2O, 500 MHz) and SEC analysis. A conversion of 84% was calculated by integrating the methyne (m, 1H, 3.90 ppm) and vinyl peaks (d, 1H, 5.65 ppm) on NIPAm to the methyne peak on PNIPAm (s, 1H, 3.80 ppm). This gave a PNIPAm $DP_{\mathrm{n,theo}}$ = 26. The aliquot was dialyzed and freeze-dried, and an absolute number average molecular weight of M_{n} = 7020 g/mol, $Đ_{\mathrm{M}}$ = 1.12, and PNIPAm DP_{n} = 11 was found by MALS.

While the quenched reaction vial was still in the heating block at 70 °C, a solution of EDC (28.7 mg, 0.150 mmol) and DMAP (2.44 mg, 0.0200 mmol, 10.0 mg/mL solution in DI water) was prepared in a separate vial. The solution was placed on the heating block for 10 min to reach 70 °C and subsequently added to the reaction vessel. After 30 min, ethylenediamine (2.16 mg, 0.0360 mmol) was added, and the solution was left to stir overnight at 70 °C. The amount of EDC was calculated by using 2× excess theoretical moles of acrylic acid (0.0738 mmol), calculated from the polymer composition (6 AA units) and amount of macro-CTA added (0.0123 mmol), while the amount ethylenediamine added was calculated as 0.5 equivalents of theoretical moles of AA.

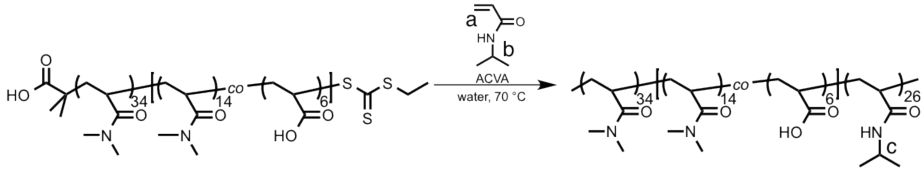

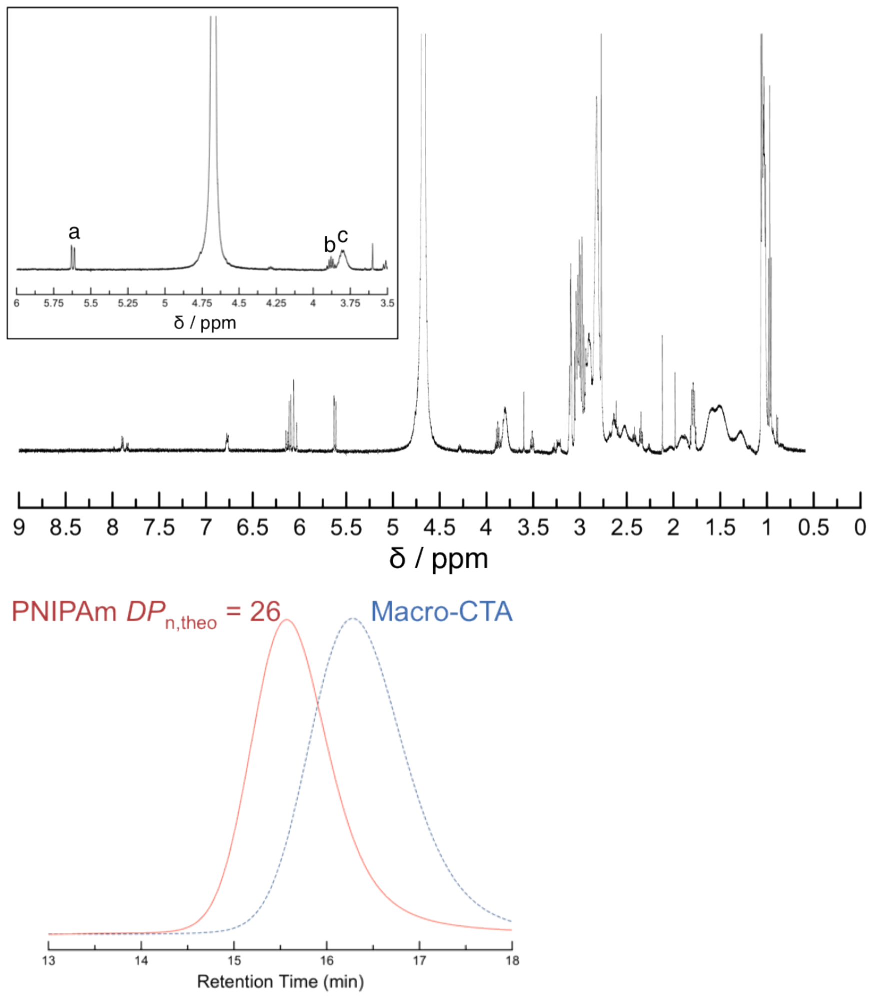

Fig. S3. Monitoring the chain extension of polyDMA$_{34}$-*b*-poly(DMA$_{14}$-*co*-AA$_6$) with NIPAm (PNIPAm $DP_{n,theo}$ = 26) by ^{1}H NMR spectroscopy (D_2O, 500 MHz) and SEC (DMAc eluent).

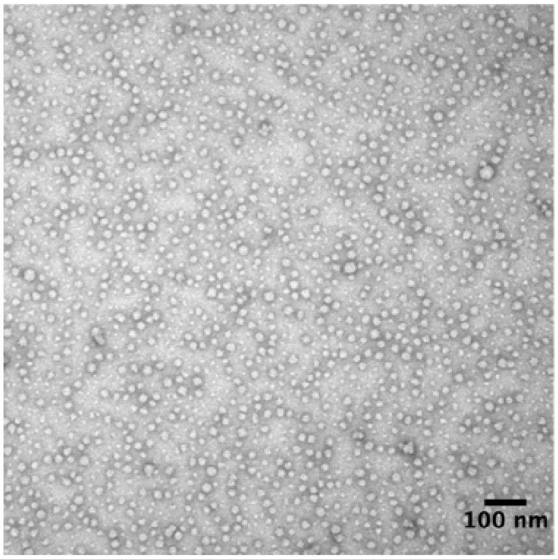

Fig. S4. TEM image of polyDMA$_{34}$-*b*-poly(DMA$_{14}$-*co*-AA$_6$)-*b*-polyNIPAm$_{26}$ crosslinked nanoparticles prior to dialysis. (scale bar = 100 nm, 2% uranyl acetate aqueous solution negative stain)

polyDMA$_{34}$-*b*-poly(DMA$_{14}$-*co*-AA$_6$)-*b*-polyNIPAm$_{38}$:

The macro-CTA polyDMA$_{34}$-*b*-poly(DMA$_{14}$-*co*-AA$_6$) (70.0 mg, 0.0123 mmol), NIPAm (56.5 mg, 0.500 mmol), ACVA (0.140 mg, 5.00 × 10^{-4} mmol, 1.00 mg/mL solution in DI water) were placed in a vial with a rubber septum to give a [NIPAm]:[CTA]:[I] ratio of [38]:[1]:[0.04]. DI water (0.718 g) was added to achieve a 15 w/w% solids concentration. The solution was deoxygenated by purging with N_2 for 30 min, then placed in a heating block at 70 °C. After 3 h, the vial was opened to the atmosphere, while still in the heating block, and subsequently stirred for 30 min to ensure reaction quenching. An aliquot (200. μL) of solution was taken for ^{1}H NMR spectroscopy (D_2O, 500 MHz) and SEC analysis. A conversion of 92% was calculated by integrating the methyne (m, 1H, 3.90 ppm) and vinyl peaks (d, 1H, 5.65 ppm) on NIPAm to the methyne peak on PNIPAm (s, 1H, 3.80 ppm). This gave a PNIPAm $DP_{n,theo}$ = 38.

While the quenched reaction vial was still in the heating block at 70 °C, a solution of EDC (28.7 mg, 0.150 mmol) and DMAP (2.44 mg, 0.0200 mmol, 10.0 mg/mL solution in DI water) was prepared in a separate vial. The solution was placed on the heating block for 10 min to reach 70 °C and subsequently added to the reaction vessel. After 30 min, ethylenediamine (2.16 mg, 0.0360 mmol) was added, and the solution was left to stir overnight at 70 °C. The amount of EDC was calculated by using 2× excess theoretical moles of acrylic acid (0.0738 mmol),

calculated from the polymer composition (6 AA units) and amount of macro-CTA added (0.0123 mmol), while the amount ethylenediamine added was calculated as 0.5 equivalents of theoretical moles of AA.

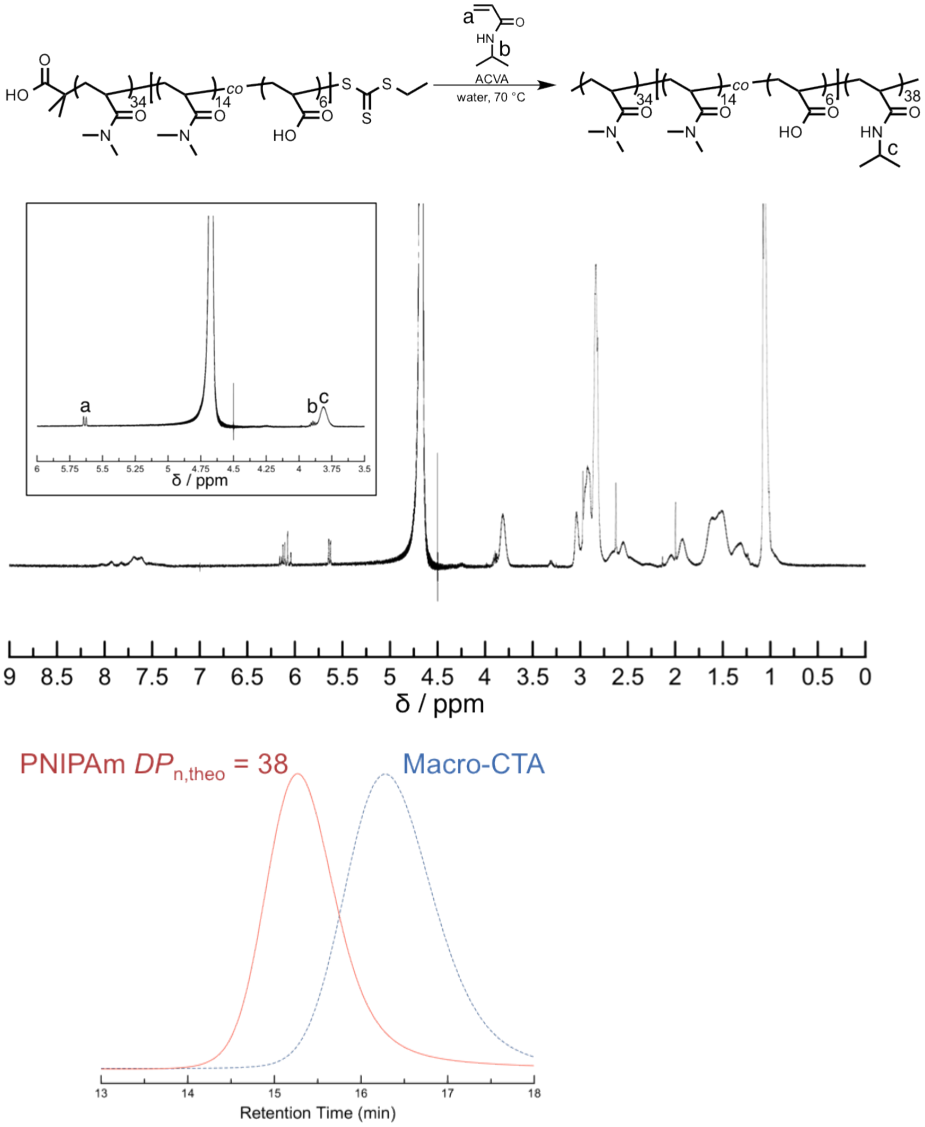

Fig. S5. Monitoring the chain extension of polyDMA$_{34}$-*b*-poly(DMA$_{14}$-*co*-AA$_6$) with NIPAm (PNIPAm $DP_{n,theo}$ = 38) by ^{1}H NMR spectroscopy (D_2O, 500 MHz) and SEC (DMAc eluent).

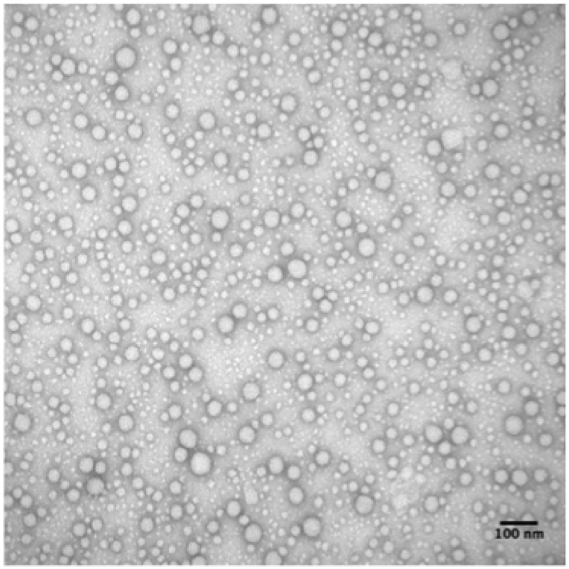

Fig. S6. TEM image of polyDMA$_{34}$-*b*-poly(DMA$_{14}$-*co*-AA$_6$)-*b*-polyNIPAm$_{38}$ crosslinked nanoparticles prior to dialysis. (scale bar = 100 nm, 2% uranyl acetate aqueous solution negative stain).

polyDMA$_{34}$-*b*-poly(DMA$_{14}$-*co*-AA$_6$)-*b*-polyNIPAm$_{49}$:

The macro-CTA polyDMA$_{34}$-*b*-poly(DMA$_{14}$-*co*-AA$_6$) (70.0 mg, 0.0123 mmol), NIPAm (67.8 mg, 0.600 mmol), ACVA (0.140 mg, 5.00×10^{-4} mmol, 1.00 mg/mL solution in DI water) were placed in a vial with a rubber septum to give a [NIPAm]:[CTA]:[I] ratio of [49]:[1]:[0.04]. DI water (0.781 g) was added to achieve a 15 w/w% solids concentration. The solution was deoxygenated by purging with N_2 for 30 min, then placed in a heating block at 70 °C. After 3 h, the vial was opened to the atmosphere, while still in the heating block, and subsequently stirred for 30 min to ensure reaction quenching. An aliquot (200. µL) of solution was taken for ^{1}H NMR spectroscopy (D_2O, 500 MHz) and SEC analysis. A conversion of 99% was calculated by integrating the methyne (m, 1H, 3.90 ppm) and vinyl peaks (d, 1H, 5.65 ppm) on NIPAm to the methyne peak on PNIPAm (s, 1H, 3.80 ppm). This gave a PNIPAm $DP_{n,theo}$ = 49. The aliquot was dialyzed and freeze-dried, and an absolute number average molecular weight of M_n = 11600 g/mol, $Đ_M$ = 1.12, and PNIPAm DP_n = 52 was found by MALS.

While the quenched reaction vial was still in the heating block at 70 °C, a solution of EDC (28.7 mg, 0.150 mmol) and DMAP (2.44 mg, 0.0200 mmol, 10.0 mg/mL solution in DI water) was prepared in a separate vial. The solution was placed on the heating block for 10 min to reach 70 °C and subsequently added to the reaction vessel. After 30 min, ethylenediamine (2.16 mg, 0.0360 mmol) was added, and the solution was left to stir overnight at 70

°C. The amount of EDC was calculated by using 2× excess theoretical moles of acrylic acid (0.0738 mmol), calculated from the polymer composition (6 AA units) and amount of macro-CTA added (0.0123 mmol), while the amount ethylenediamine added was calculated as 0.5 equivalents of theoretical moles of AA.

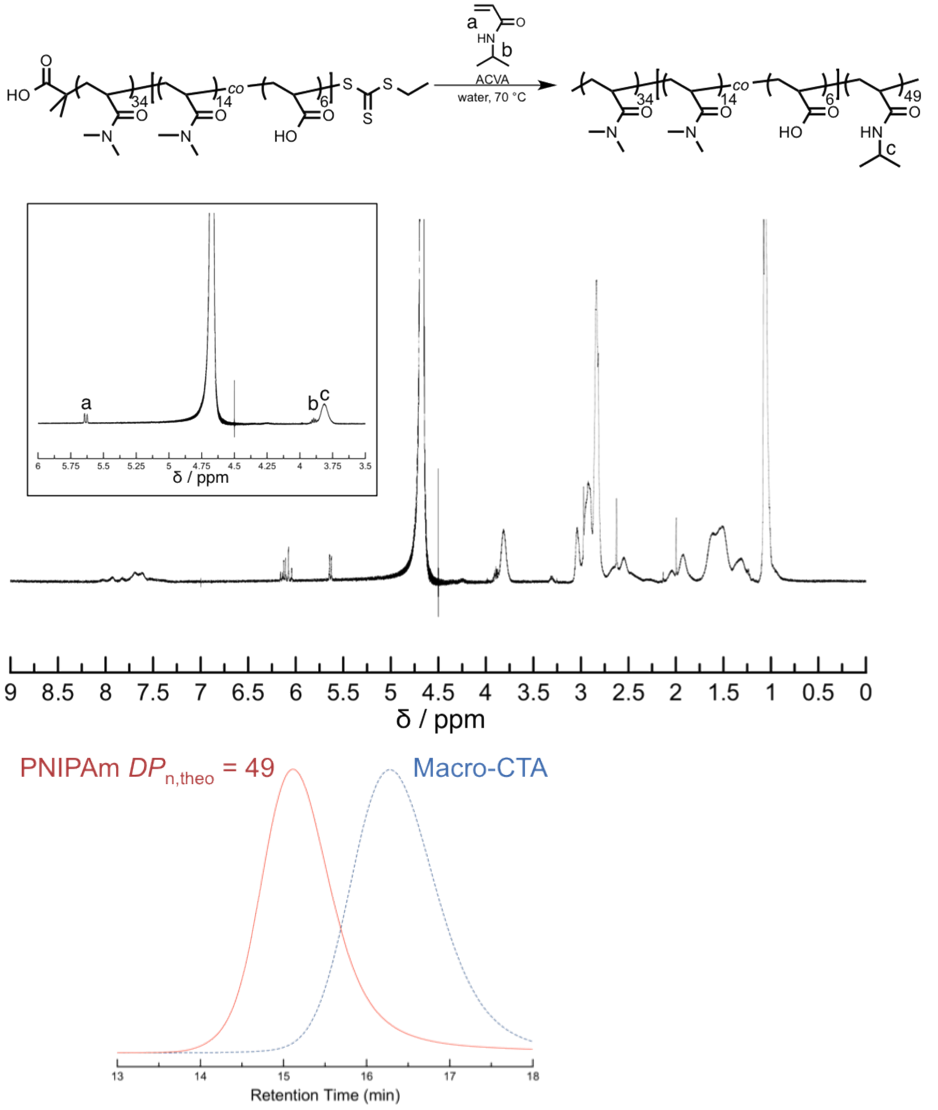

Fig. S7. Monitoring the chain extension of polyDMA$_{34}$-*b*-poly(DMA$_{14}$-*co*-AA$_6$) with NIPAm (PNIPAm $DP_{n,theo}$ = 49) by ^{1}H NMR spectroscopy (D_2O, 500 MHz) and SEC (DMAc eluent).

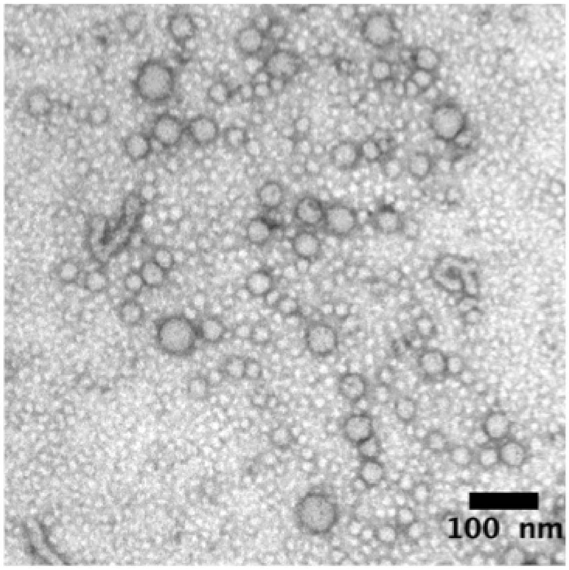

Fig. S8. TEM image of polyDMA$_{34}$-*b*-poly(DMA$_{14}$-*co*-AA$_6$)-*b*-polyNIPAm$_{49}$ crosslinked nanoparticles prior to dialysis. (scale bar = 100 nm, 2% uranyl acetate aqueous solution negative stain).

polyDMA$_{34}$-*b*-poly(DMA$_{14}$-*co*-AA$_6$)-*b*-polyNIPAm$_{61}$:

The macro-CTA polyDMA$_{34}$-*b*-poly(DMA$_{14}$-*co*-AA$_6$) (70.0 mg, 0.0123 mmol), NIPAm (90.4 mg, 0.800 mmol), ACVA (0.140 mg, 5.00 × 10^{-4} mmol, 1.00 mg/mL solution in DI water) were placed in a vial with a rubber septum to give a [NIPAm]:[CTA]:[I] ratio of [65]:[1]:[0.04]. DI water (0.910 g) was added to achieve a 15 w/w% solids concentration. The solution was deoxygenated by purging with N_2 for 30 min, then placed in a heating block at 70 °C. After 3 h, the vial was opened to the atmosphere, while still in the heating block, and subsequently stirred for 30 min to ensure reaction quenching. An aliquot (200. µL) of solution was taken for ^{1}H NMR spectroscopy (D_2O, 500 MHz) and SEC analysis. A conversion of 94% was calculated by integrating the methyne (m, 1H, 3.90 ppm) and vinyl peaks (d, 1H, 5.65 ppm) on NIPAm to the methyne peak on PNIPAm (s, 1H, 3.80 ppm). This gave a PNIPAm $DP_{n,theo}$ = 61.

While the quenched reaction vial was still in the heating block at 70 °C, a solution of EDC (28.7 mg, 0.150 mmol) and DMAP (2.44 mg, 0.0200 mmol, 10.0 mg/mL solution in DI water) was prepared in a separate vial. The solution was placed on the heating block for 10 min to reach 70 °C and subsequently added to the reaction vessel. After 30 min, ethylenediamine (2.16 mg, 0.0360 mmol) was added, and the solution was left to stir overnight at 70 °C. The amount of EDC was calculated by using 2× excess theoretical moles of acrylic acid (0.0738 mmol),

calculated from the polymer composition (6 AA units) and amount of macro-CTA added (0.0123 mmol), while the amount ethylenediamine added was calculated as 0.5 equivalents of theoretical moles of AA.

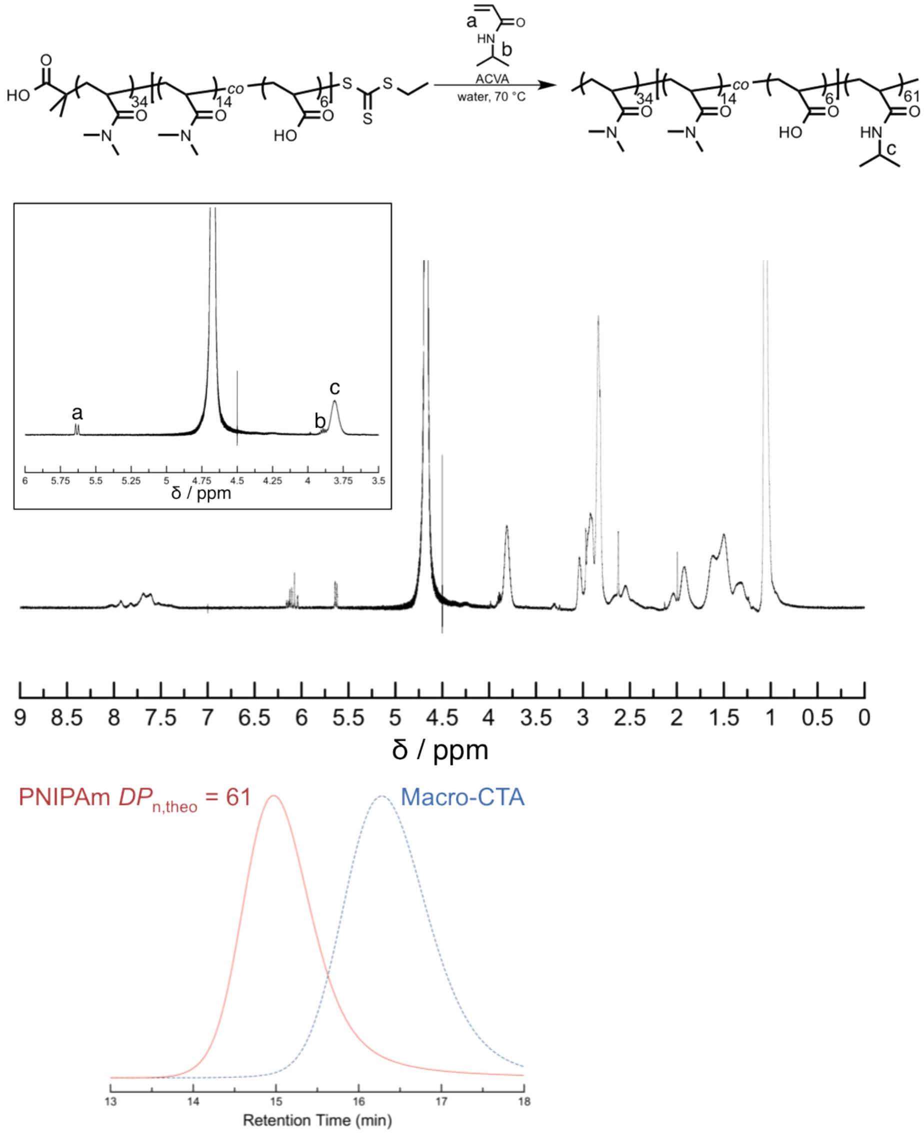

Fig. S9. Monitoring the chain extension of polyDMA$_{34}$-*b*-poly(DMA$_{14}$-*co*-AA$_6$) with NIPAm (PNIPAm $DP_{n,theo}$ = 61) by ^{1}H NMR spectroscopy (D_2O, 500 MHz) and SEC (DMAc eluent).

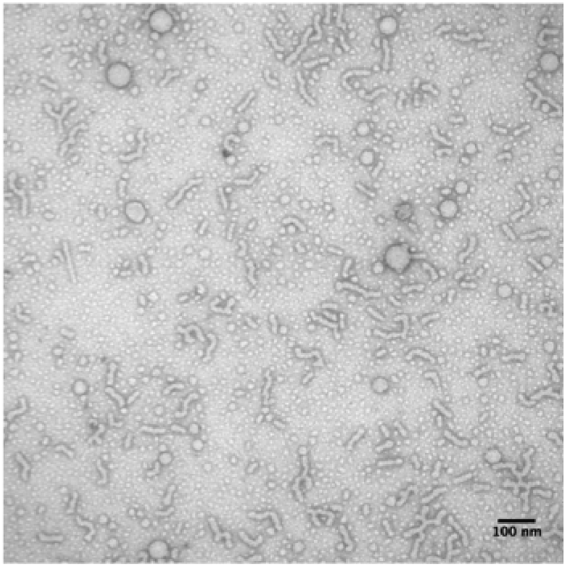

Fig. S10. TEM image of polyDMA$_{34}$-*b*-poly(DMA$_{14}$-*co*-AA$_6$)-*b*-polyNIPAm$_{61}$ crosslinked nanoparticles prior to dialysis. (scale bar = 100 nm, 2% uranyl acetate aqueous solution negative stain).

polyDMA$_{34}$-*b*-poly(DMA$_{14}$-*co*-AA$_6$)-*b*-polyNIPAm$_{73}$:

The macro-CTA polyDMA$_{34}$-*b*-poly(DMA$_{14}$-*co*-AA$_6$) (70.0 mg, 0.0123 mmol), NIPAm (102 mg, 0.900 mmol), ACVA (0.140 mg, 5.00×10^{-4} mmol, 1.00 mg/mL solution in DI water) were placed in a vial with a rubber septum to give a [NIPAm]:[CTA]:[I] ratio of [73]:[1]:[0.04]. DI water (0.975 g) was added to achieve a 15 w/w% solids concentration. The solution was deoxygenated by purging with N_2 for 30 min, then placed in a heating block at 70 °C. After 3 h, the vial was opened to the atmosphere, while still in the heating block, and subsequently stirred for 30 min to ensure reaction quenching. An aliquot (200. μL) of solution was taken for ^{1}H NMR spectroscopy (D_2O, 500 MHz) and SEC analysis. A conversion of >99% was calculated by integrating the methyne (m, 1H, 3.90 ppm) and vinyl peaks (d, 1H, 5.65 ppm) on NIPAm to the methyne peak on PNIPAm (s, 1H, 3.80 ppm). This gives a PNIPAm $DP_{n,theo}$ = 73. The aliquot was dialyzed and freeze-dried, and an absolute number average molecular weight of M_n = 17100 g/mol, $Đ_M$ = 1.07, and PNIPAm DP_n = 101 was found by MALS.

While the quenched reaction vial was still in the heating block at 70 °C, a solution of EDC (28.7 mg, 0.150 mmol) and DMAP (2.44 mg, 0.0200 mmol, 10.0 mg/mL solution in DI water) was prepared in a separate vial. The solution was placed on the heating block for 10 min to reach 70 °C and subsequently added to the reaction vessel. After 30 min, ethylenediamine (2.16 mg, 0.0360 mmol) was added, and the solution was left to stir overnight at 70 °C. The amount of EDC was calculated by using 2× excess theoretical moles of acrylic acid (0.0738 mmol),

calculated from the polymer composition (6 AA units) and amount of macro-CTA added (0.0123 mmol), while the amount ethylenediamine added was calculated as 0.5 equivalents of theoretical moles of AA.

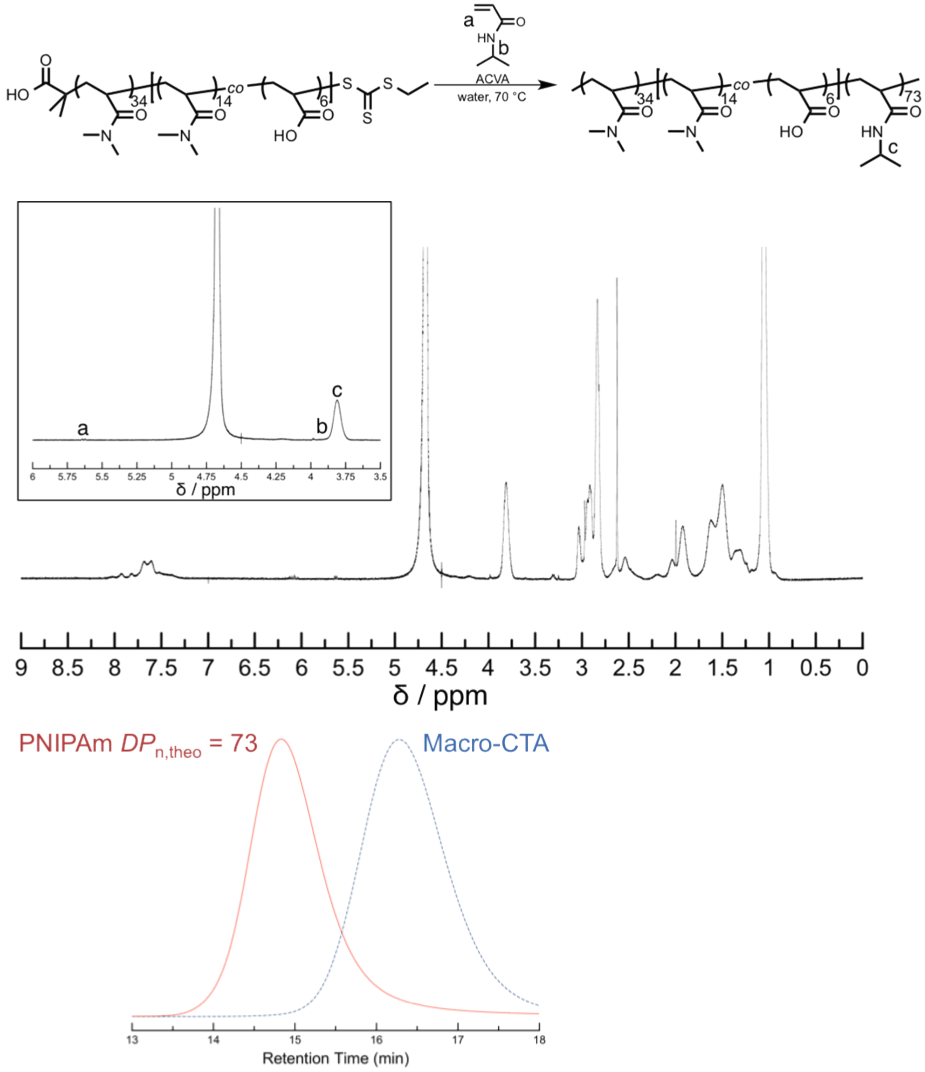

Fig. S11. Monitoring the chain extension of polyDMA$_{34}$-*b*-poly(DMA$_{14}$-*co*-AA$_6$) with NIPAm (PNIPAm $DP_{n,theo}$ = 73) by ^{1}H NMR spectroscopy (D_2O, 500 MHz) and SEC (DMAc eluent).

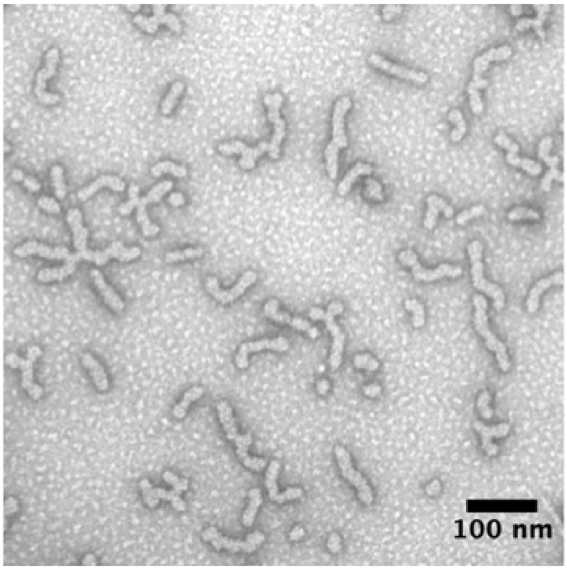

Fig. S12. TEM image of polyDMA$_{34}$-*b*-poly(DMA$_{14}$-*co*-AA$_6$)-*b*-polyNIPAm$_{73}$ crosslinked nanoparticles prior to dialysis. (scale bar = 100 nm, 2% uranyl acetate aqueous solution negative stain).

polyDMA$_{34}$-*b*-poly(DMA$_{14}$-*co*-AA$_6$)-*b*-polyNIPAm$_{98}$:

The macro-CTA polyDMA$_{34}$-*b*-poly(DMA$_{14}$-*co*-AA$_6$) (70.0 mg, 0.0123 mmol), NIPAm (136 mg, 1.20 mmol), ACVA (0.140 mg, 5.00 × 10^{-4} mmol, 1.00 mg/mL solution in DI water) were placed in a vial with a rubber septum to give a [NIPAm]:[CTA]:[I] ratio of [98]:[1]:[0.04]. DI water (1.17 g) was added to achieve a 15 w/w% solids concentration. The solution was deoxygenated by purging with N_2 for 30 min, then placed in a heating block at 70 °C. After 3 h, the vial was opened to the atmosphere, while still in the heating block, and subsequently stirred for 30 min to ensure reaction quenching. An aliquot (200. µL) of solution was taken for ^{1}H NMR spectroscopy (D_2O, 500 MHz) and SEC analysis. A conversion of >99% was calculated by integrating the methyne (m, 1H, 3.90 ppm) and vinyl peaks (d, 1H, 5.65 ppm) on NIPAm to the methyne peak on PNIPAm (s, 1H, 3.80 ppm). This gives a PNIPAm $DP_{n,theo}$ = 98.

While the quenched reaction vial was still in the heating block at 70 °C, a solution of EDC (28.7 mg, 0.150 mmol) and DMAP (2.44 mg, 0.0200 mmol, 10.0 mg/mL solution in DI water) was prepared in a separate vial. The solution was placed on the heating block for 10 min to reach 70 °C and subsequently added to the reaction vessel. After 30 min, ethylenediamine (2.16 mg, 0.0360 mmol) was added, and the solution was left to stir overnight at 70 °C. The amount of EDC was calculated by using 2× excess theoretical moles of acrylic acid (0.0738 mmol),

calculated from the polymer composition (6 AA units) and amount of macro-CTA added (0.0123 mmol), while the amount ethylenediamine added was calculated as 0.5 equivalents of theoretical moles of AA.

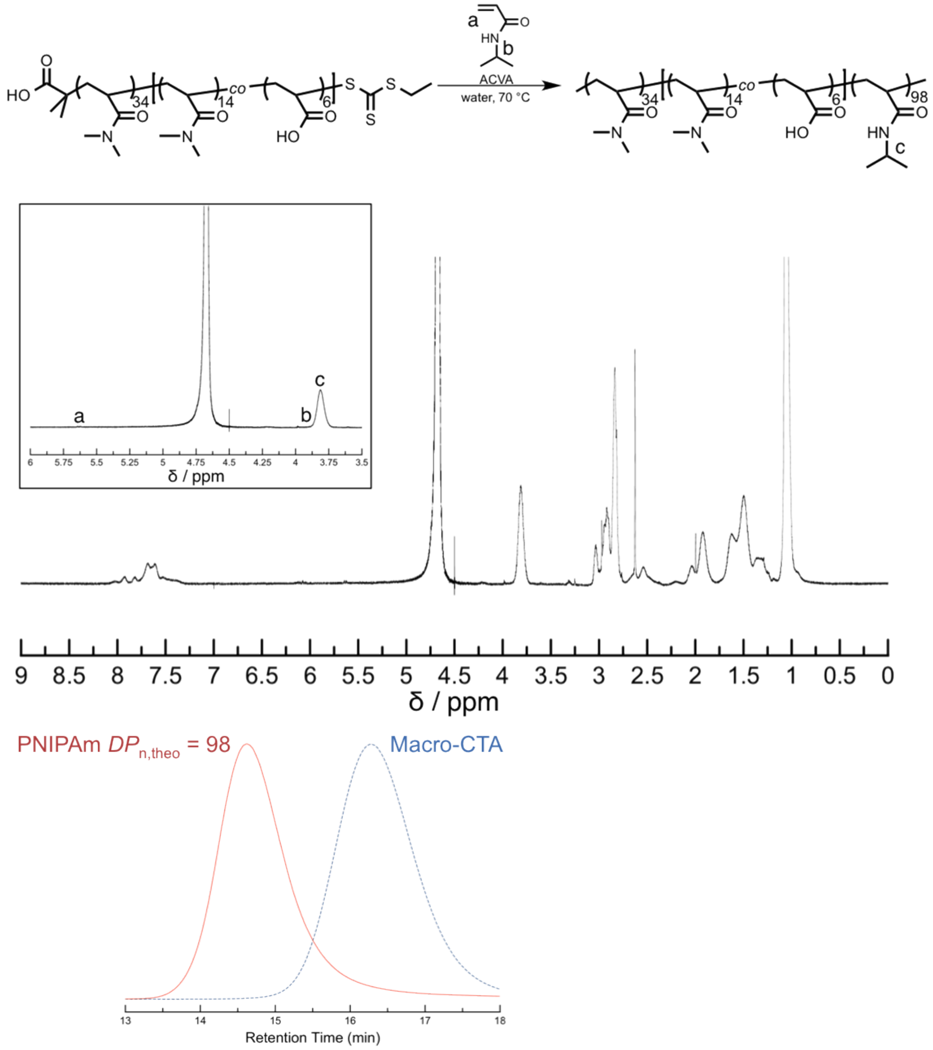

Fig. S13. Monitoring the chain extension of polyDMA$_{34}$-*b*-poly(DMA$_{14}$-*co*-AA$_6$) with NIPAm (PNIPAm $DP_{n,theo}$ = 98) by ^{1}H NMR spectroscopy (D_2O, 500 MHz) and SEC (DMAc eluent).

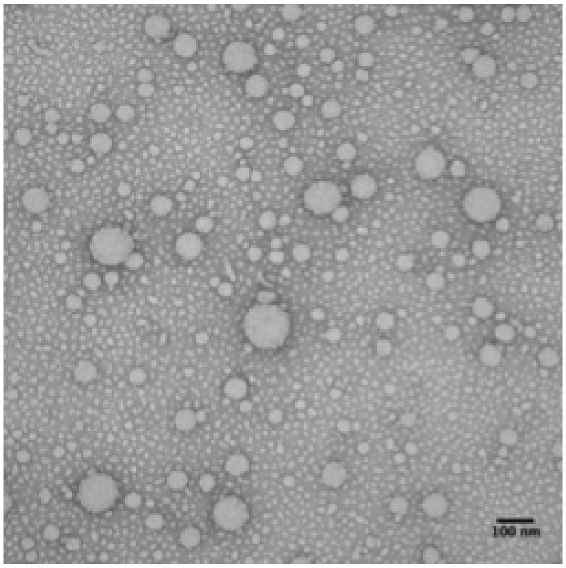

Fig. S14 TEM image of polyDMA$_{34}$-*b*-poly(DMA$_{14}$-*co*-AA$_6$)-*b*-polyNIPAm$_{98}$ crosslinked nanoparticles* prior to dialysis. (scale bar = 100 nm, 2% uranyl acetate aqueous solution negative stain).

*Larger particle sizes are attributed to vesicle-like morphologies due to similar sizes compared to a PNIPAm DP_n = 137 and absence of these size distributions in lower PNIPAm DP_n TEM imaging.

polyDMA$_{34}$-*b*-poly(DMA$_{14}$-*co*-AA$_6$)-*b*-polyNIPAm$_{121}$:

The macro-CTA polyDMA$_{34}$-*b*-poly(DMA$_{14}$-*co*-AA$_6$) (70.0 mg, 0.0123 mmol), NIPAm (168 mg, 1.49 mmol), ACVA (0.140 mg, 5.00 × 10^{-4} mmol, 1.00 mg/mL solution in DI water) were placed in a vial with a rubber septum to give a [NIPAm]:[CTA]:[I] ratio of [121]:[1]:[0.04]. DI water (1.35 g) was added to achieve a 15 w/w% solids concentration. The solution was deoxygenated by purging with N_2 for 30 min, then placed in a heating block at 70 °C. After 3 h, the vial was opened to the atmosphere, while still in the heating block, and subsequently stirred for 30 min to ensure reaction quenching. An aliquot (200. µL) of solution was taken for ^{1}H NMR spectroscopy (D_2O, 500 MHz) and SEC analysis. A conversion of >99% was calculated by integrating the methyne (m, 1H, 3.90 ppm) and vinyl peaks (d, 1H, 5.65 ppm) on NIPAm to the methyne peak on PNIPAm (s, 1H, 3.80 ppm). This gives a PNIPAm $DP_{n,theo}$ = 121. The aliquot was dialyzed and freeze-dried, and an absolute number average molecular weight of M_n = 21200 g/mol, $Đ_M$ = 1.06, and PNIPAm DP_n = 137 was found by MALS.

While the quenched reaction vial was still in the heating block at 70 °C, a solution of EDC (28.7 mg, 0.150 mmol) and DMAP (2.44 mg, 0.0200 mmol, 10.0 mg/mL solution in DI water) was prepared in a separate vial. The

solution was placed on the heating block for 10 min to reach 70 °C and subsequently added to the reaction vessel. After 30 min, ethylenediamine (2.16 mg, 0.0360 mmol) was added, and the solution was left to stir overnight at 70 °C. The amount of EDC was calculated by using 2× excess theoretical moles of acrylic acid (0.0738 mmol), calculated from the polymer composition (6 AA units) and amount of macro-CTA added (0.0123 mmol), while the amount ethylenediamine added was calculated as 0.5 equivalents of theoretical moles of AA.

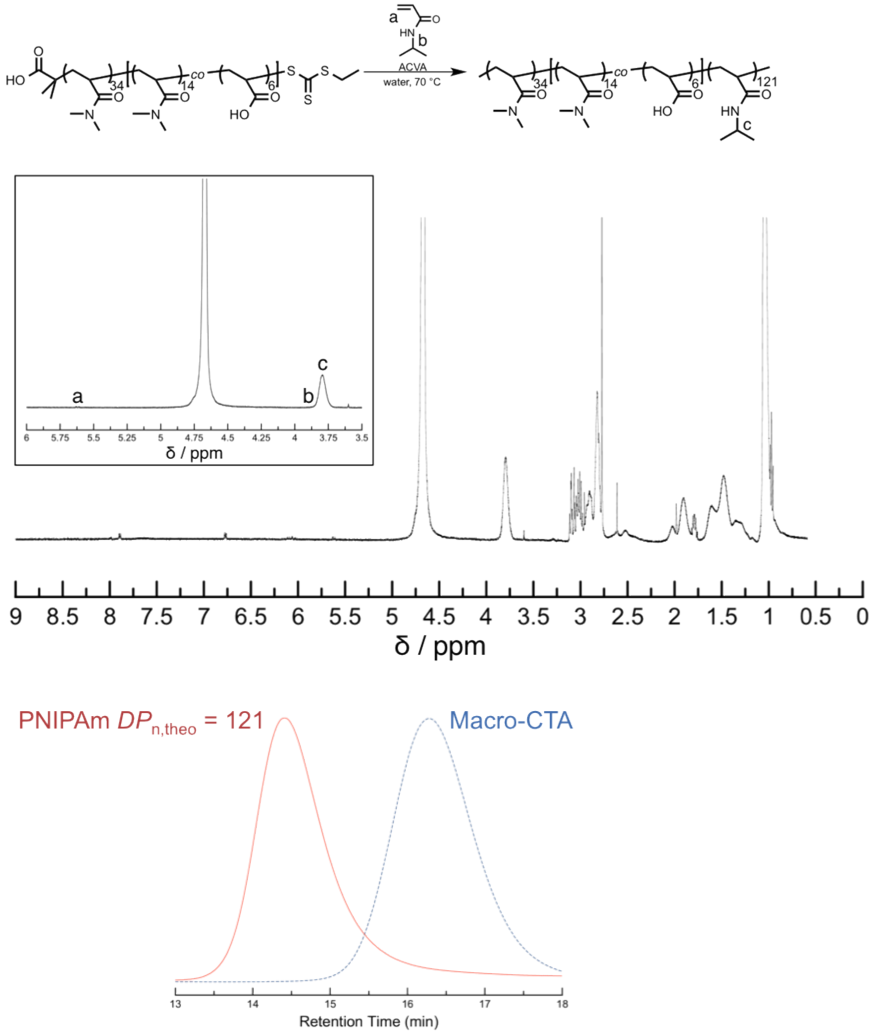

Fig. S15. Monitoring the chain extension of polyDMA$_{34}$-*b*-poly(DMA$_{14}$-*co*-AA$_6$) with NIPAm (PNIPAm $DP_{n,theo}$ = 121) by ^{1}H NMR spectroscopy (D_2O, 500 MHz) and SEC (DMAc eluent).

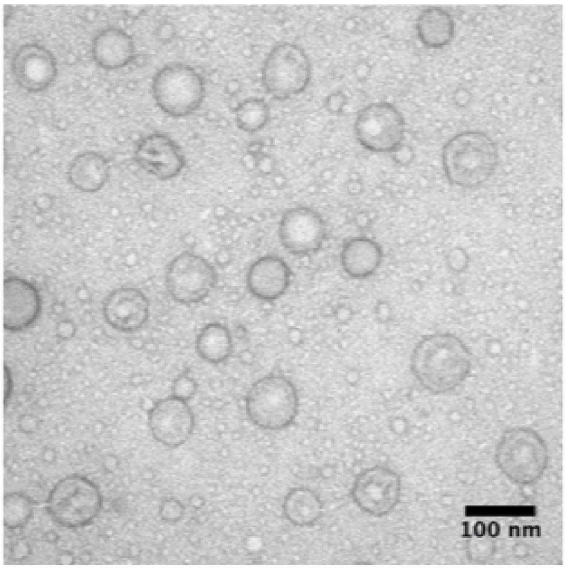

Fig. S16. TEM image polyDMA$_{34}$-*b*-poly(DMA$_{14}$-*co*-AA$_6$)-*b*-polyNIPAm$_{121}$ crosslinked nanoparticles prior to dialysis. (scale bar = 100 nm, 2% uranyl acetate aqueous solution negative stain).

Kinetics of poly(*N*-isopropylacrylamide) chain-extension

The macro-CTA polyDMA$_{34}$-*b*-poly(DMA$_{14}$-*co*-AA$_6$) (350. mg, 0.0615 mmol), NIPAm (841 mg, 7.45 mmol), and ACVA (0.700 mg, 2.50×10^{-3} mmol, 1.00 mg/mL solution in DI water) ([NIPAm]:[CTA]:[I] ratio of [121]:[1]:[0.04]) were placed in a vial. DI water (6.76 g) was added to achieve a 15 w/w% solids concentration with 1,3,5-trioxane (215 mg) as an internal standard. The solution was divided into six vials containing 1.20 mL each. The vials were deoxygenated by purging with N_2 for 30 min then placed in a heating block at 70 °C. Every 30 min a vial was removed from the heat, quenched by opening to the atmosphere in a cold water bath, and analyzed by ^{1}H NMR spectroscopy ($CDCl_3$, 500 MHz) and SEC for conversion and molecular weight.

Additional Figures

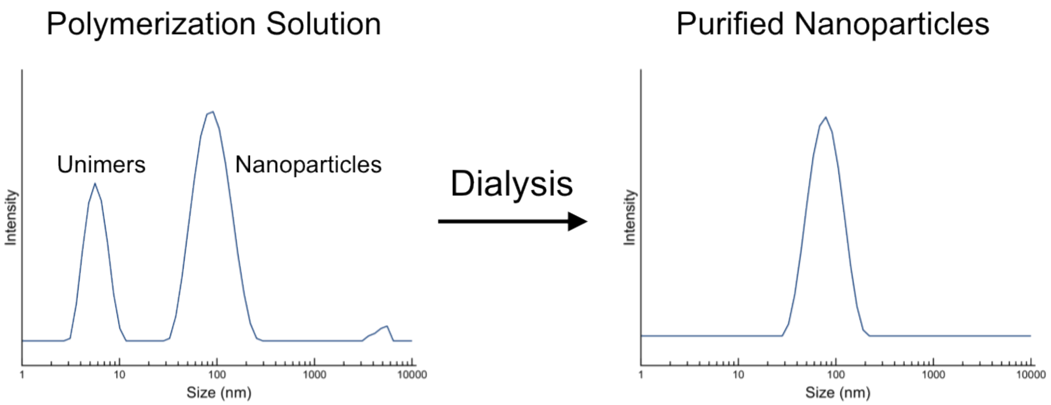

Fig. S17 DLS measurements following PITSA of polyDMA$_{34}$-*b*-poly(DMA$_{14}$-*co*-AA$_6$)-*b*-polyNIPAm$_{101}$ revealed unimer and nanoparticle size distributions; however, dialysis of the polymerization solution yielded purified nanoparticles.

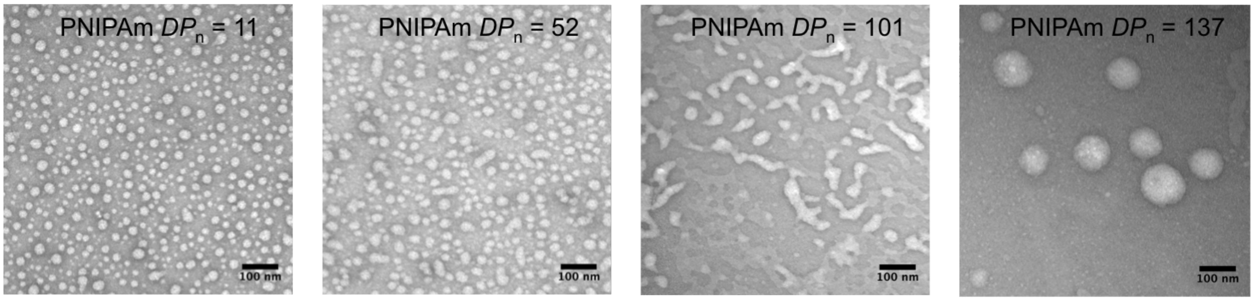

Fig. S18. TEM images of polyDMA$_{34}$-*b*-poly(DMA$_{14}$-*co*-AA$_6$)-*b*-polyNIPAm$_n$ following purification by dialysis. (scale bar = 100 nm, 2% uranyl acetate aqueous solution negative stain).

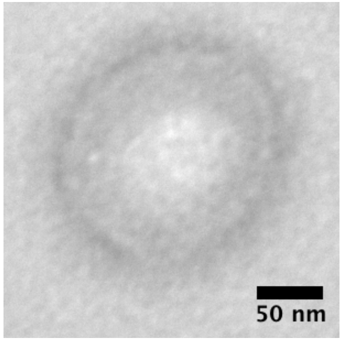

Fig. S19. Transmission electron microscopy with an osmium tetroxide positive stain showed the vesicle nanoparticle bilayer composed of polyDMA$_{34}$-*b*-poly(DMA$_{14}$-*co*-AA$_6$)-*b*-polyNIPAm$_{137}$.

References:

(1) Li, H.; Bapat, A. P.; Li, M.; Sumerlin, B. S. *Polym. Chem.* **2011**, *2*, 323-327.
